# Impact of Caries-Related Pulp Involvement on Oral Health-Related Quality of Life Among Turkish Preschool Children: A Cross-Sectional Study

**DOI:** 10.3290/j.ohpd.c_2839

**Published:** 2026-09-08

**Authors:** Gülser Kılınç, Gülçin Bulut, Saime Esin Güney

**Affiliations:** a Gülser Kılınç Professor, Department of Pediatric Dentistry, Faculty of Dentistry, Dokuz Eylul University, İzmir 35340, Turkey; conceived the study idea and hypothesis, contributed to the data collection, and led the writing and manuscript preparation; b Gülçin Bulut Associate Professor, Department of Pediatric Dentistry, Faculty of Dentistry, Dokuz Eylul University, İzmir 35340, Turkey; conceived the study idea and hypothesis; contributed to data collection; led the writing and manuscript preparation; c Saime Esin Güney Doctor, Department of Pediatric Dentistry, Faculty of Dentistry, Dokuz Eylul University, İzmir 35340, Turkey; performed the data collection and statistical analysis

**Keywords:** early childhood caries, Early Childhood Oral Health Impact Scale, oral health–related quality of life, pufa index, pulpal involvement

## Abstract

**Objective:**

To assess the impact of early childhood caries (ECC) and caries-related pulpal involvement on the oral health–related quality of life (OHRQoL) of Turkish preschool children aged 12 to 72 months using the Early Childhood Oral Health Impact Scale (ECOHIS).

**Materials and Methods:**

A total of 354 children aged 12 to 72 months with ECC and their parents/caregivers participated in this cross-sectional study. Oral examinations were performed using the decayed, missing, and filled teeth (dmft) index and the pufa index, which records pulpal involvement (P/p), ulceration (U/u), fistula (F/f), and abscess (A/a), followed by a structured interview with parents/caregivers to complete the validated Turkish version of the 13-item ECOHIS questionnaire.

**Results:**

The prevalence and mean scores of ECC (dmft) and pufa were 77.1%/5.77 ± 4.78 and 50.8%/1.54 ± 2.11, respectively. Increasing severity of ECC was significantly associated with poorer OHRQoL (*P* < 0.001). Children with pufa > 0 had significantly higher ECOHIS scores (15.64 ± 7.69) compared to those with pufa = 0 (7.16 ± 6.67) (*P* < 0.001). Multiple linear regression analysis revealed that pulpal involvement was the strongest predictor of OHRQoL deterioration (B = 7.656, 95% CI 5.790 to 9.523, *P* < 0.001), explaining 28% of the variance in ECOHIS scores.

**Conclusion:**

ECC and pulpal involvement adversely affected the OHRQoL of Turkish preschool children and their families. The pufa index provides a clinically meaningful complement to the dmft index in assessing the true burden of untreated caries.

Although there have been significant advancements in the prevention and treatment of cavities in primary teeth in recent years, tooth decay remains a significant issue and the most prevalent disease among children, mostly in low- and middle-income families, largely due to high consumption of cariogenic foods, poor oral hygiene routines, and a lack of regular dental visits.^5,7
^ The term ‘early childhood caries’ (ECC) encompasses any form of dental caries affecting the primary dentition in children aged 71 months and younger.^4^ If ECC in primary teeth is left untreated, it can lead to complications such as toothache, dental fistulas, abscesses, ulcers, and infections.^15,16
^


Numerous studies have shown that oral health significantly affects a child’s overall health and quality of life.^4,22,14
^ Poor oral health can lead to malnutrition, sleep disruption, school absences, and behavioural issues, negatively impacting growth and development.^4,22
^


Oral health–related quality of life (OHRQoL) is a multidimensional construct that reflects the impact of oral conditions on an individual’s functional, psychological, and overall well-being.^26^ Today, assessing the influence of preschool oral conditions on children’s quality of life is increasingly important.^4,22
^ The Early Childhood Oral Health Impact Scale (ECOHIS), based on parental or caregiver responses, was developed to assess OHRQoL in preschool children^14^ and has been widely validated in national and international studies.^8,19,24
^


According to World Health Organization (WHO) criteria, the DMFT/dmft (decayed, missing, and filled teeth) index assesses caries in permanent and primary teeth, respectively.^29^ However, it does not reflect advanced stages of decay. To address this limitation, Monse et al^16^ introduced the PUFA/pufa index, which records pulpal involvement (P/p), ulceration (U/u), fistula (F/f), and abscess (A/a), providing a more detailed assessment of caries severity. The reported prevalence of PUFA/pufa in primary and permanent teeth varies considerably across countries, ranging from 4% to 85%. The prevalence of PUFA in permanent teeth was observed to be significantly lower than that in primary teeth.^16,27
^ Vasavan and Retnakumari^27^ reported a PUFA prevalence of 43.1% in primary teeth and 4.1% in permanent teeth, with the ‘p’ component being the most common among their study population.

The aim of this study is to assess the oral health status, oral health behaviors, and feeding habits of children aged 12 to 72 months, along with their sociodemographic characteristics, and to examine the impact of ECC and PUFA/pufa on OHRQoL using the ECOHIS.

## Materials and methods

### Study Population and Design

This descriptive, cross-sectional study was conducted on patients aged 12 to 72 months who attended the Pediatric Dentistry clinics at the Faculty of Dentistry, Dokuz Eylul University, İzmir, Turkey, between April 2023 and January 2024. Over the course of the study, 644 Turkish children aged 12 to 72 months attended the Pediatric Dental Clinic. Following the application of the exclusion criteria, 393 children met the eligibility criteria and were included in the study. Of these 393 eligible children, 354 completed the study (90%), while the remainder were excluded owing to incomplete questionnaires. Before any children were enrolled, a cover letter and informed consent form were provided to the parents of eligible participants, clearly explaining the study objectives and procedures. Children who were uncooperative, medically compromised, accompanied by someone other than their parents or guardians, whose parents or guardians were unwilling to participate, and whose questionnaires were incomplete were excluded from the study.

G*Power software (version 3.1.9.4) was used to determine the necessary sample size, which was based on the mean ± standard deviation (SD) from an earlier study with a power of 90%, and a Type I error rate of 5%.^18^ This calculation indicated that 218 participants were needed. This target number was surpassed in the study.

This study received ethical approval from the Dokuz Eylul University Research Ethics Committee (protocol number 2023/10-13). The parents or guardians of all participating children provided written informed consent prior to enrollment in the study.

### Data Collection

Data were collected using a structured demographic survey and the ECOHIS questionnaire. The participating patients underwent clinical examinations.

Sociodemographic information about the children was gathered from one parent, including age, sex, parental education level, family income, number of children, frequency of toothbrushing, frequency of dental visits, whether the children were breastfed or bottle-fed while sleeping, and whether they were given sweetened milk.

### ECOHIS Questionnaire

The Turkish-validated ECOHIS was administered to the same parent to assess their child’s OHRQoL.^19^ The ECOHIS is divided into two sections and comprises 13 questions. The first section, containing nine questions, is the Child Impact Section (CIS), and the second, composed of four questions, is the Family Impact Section (FIS). The CIS is organised into four subscales, namely child symptoms, child functions, child psychology, and child self-image/social interaction, while the FIS encompasses two subscales: parent distress and family function. Each item is rated from 0 to 5, where 0 = never, 1 = rarely, 2 = sometimes, 3 = often, 4 = very often, and 5 = don’t know. The CIS and FIS response codes are summed to determine the child’s ECOHIS score, with ‘don’t know’ responses classified as missing data. With a CIS score of 0 to 36 and a FIS score of 0 to 16, the overall score ranges between 0 and 52. Higher scores indicate a poorer OHRQoL and a greater adverse effect on oral health.

### Clinical Assessment

The dental examinations were conducted with children seated in a dental chair, using a mirror and probe, without radiographic evaluation. Before the examination, the teeth were cleaned and dried with an air spray. For primary dentition, the dmft index was used to determine caries status in accordance with WHO guidelines.^29^ Diagnostic criteria for ECC and severe ECC (S-ECC) were established in accordance with the American Academy of Pediatric Dentistry (AAPD) guidelines and the corresponding index. ECC was defined as the presence of one or more decayed, missing due to caries, or filled surfaces in any primary tooth of a child younger than 72 months. S-ECC was diagnosed when the dmft score reached ≥ 4 at age 3, ≥ 5 at age 4, or ≥ 6 at age 5.^3^


All examinations were performed by two calibrated investigators (GK and GB) who had completed standardised training in caries and pufa assessment. Training involved examining 20 age-matched patients who visited the clinic 14 days before the study; these individuals were excluded from the study sample. The same patients were re-examined by both investigators 7 days later to assess consistency. Kappa (κ) statistics were used to determine intra- and inter-examiner reliability. The inter-examiner κ value for the pufa index was 0.82, while that for the dmft index was 0.84. Intra-examiner reliability was also high, with κ values of 0.88 and 0.86 for GK and GB, respectively, across both indices.

### Statistical Analysis

All statistical analyses were carried out using SPSS version 22.0 (IBM, Armonk, NY, USA). Differences between groups were assessed using a Kruskal-Wallis test, and pairwise post hoc comparisons were carried out with Bonferroni correction. Relationships between continuous variables were evaluated using a Spearman rank correlation test, whereas associations between categorical variables were examined with a chi-square test. Multiple linear regression analysis was employed to investigate the associations between caries-related variables (dt, mt, and pufa) and ECOHIS scores. Regression results were reported as *P* values, 95% confidence intervals (CIs), standardised coefficients (β), and unstandardised coefficients (B). Statistical significance was set at *P* < 0.05.

Severity of caries was defined as no caries, ECC = 0; caries, ECC > 0; non-severe ECC, ECC > 0 + S-ECC = 0; and severe ECC, S-ECC > 0. Carious conditions were grouped as Group 1, no caries (ECC = 0); Group 2, caries without pufa (ECC > 0 + pufa = 0); and Group 3, caries with pufa (pufa > 0).

## Results

A total of 354 children were enrolled in the study, corresponding to a response rate of 90%. Incomplete questionnaire completion was the primary reason for participant dropout. The final study population consisted of 49.4% boys (n = 175) and 50.6% girls (n = 179) with a mean age of 3.87 ± 1.16 years. The numerical distribution of the children and their family characteristics by age group is presented in Table 1. The prevalences and mean scores of ECC (dmft) and pufa were 77.1%/5.77 ± 4.78 and 50.8%/1.54 ± 2.11, respectively. Four children were found to have a dmft score of 20, all of which were due to decay (d). Half of the study group had at least one pufa score. The highest pufa score was 10, recorded in three children. The most common pufa component was pulpal involvement (47.2%), followed by ulceration (7.9%). A significant positive correlation was observed between dmft and pufa scores (r = 0.676, *P* = 0.0001). Distributions of ECC and pufa are shown in Table 2.

**Table 1 Table1:** Distribution of children and family characteristics according to age group

Characteristic		1 year (12–23 mo), n (%)	2 years (24–35 mo), n (%)	3 years (36–47 mo), n (%)	4 years (48–59 mo), n (%)	5 years (60–72 mo), n (%)	Total (N = 354)	*P* value
Sex	Girl	5 (35.7)	20 (50.0)	38 (62.3)	50 (49.5)	66 (47.8)	179 (50.6)	0.281*
	Boy	9 (64.3)	20 (50.0)	23 (37.7)	51 (50.5)	72 (52.2)	175 (49.4)	
Educational level of parent	None	0 (0.0)	2 (5.0)	2 (3.3)	3 (3.0)	3 (2.2)	10 (2.8)	0.457^†^
	Primary–secondary	1 (7.1)	5 (12.5)	11 (18.0)	23 (22.8)	30 (21.7)	70 (19.8)	
	High school	4 (28.6)	11 (27.5)	17 (27.9)	37 (36.6)	53 (38.4)	122 (34.5)	
	University	9 (64.3)	22 (55.0)	31 (50.8)	38 (37.6)	52 (37.7)	152 (42.9)	
Family income	Low	5 (35.7)	19 (47.5)	23 (37.7)	40 (39.6)	72 (52.2)	159 (44.9)	0.322^†^
	Medium	3 (21.4)	12 (30.0)	18 (29.5)	36 (35.6)	35 (25.4)	104 (29.4)	
	High	6 (42.9)	9 (22.5)	20 (32.8)	25 (24.8)	31 (22.5)	91 (25.7)	
Number of children	1	9 (64.3)	22 (55.0)	29 (47.5)	38 (37.6)	35 (25.4)	133 (37.6)	0.007^†^
	2	5 (35.7)	14 (35.0)	25 (41.0)	48 (47.5)	71 (51.4)	163 (46.0)	
	3	0 (0.0)	4 (10.0)	7 (11.5)	14 (13.9)	26 (18.8)	51 (14.4)	
	4 or more	0 (0.0)	0 (0.0)	0 (0.0)	1 (1.0)	6 (4.3)	7 (2.0)	
Frequency of toothbrushing	Once a day	3 (21.4)	20 (50.0)	22 (36.1)	45 (44.6)	70 (50.7)	160 (45.2)	< 0.001^†^
	Twice a day	3 (21.4)	8 (20.0)	31 (50.8)	34 (33.7)	33 (23.9)	109 (30.8)	
	Irregular	8 (57.1)	12 (30.0)	8 (13.1)	22 (21.8)	35 (25.4)	85 (24.0)	
Dental visits	Yes	3 (21.4)	21 (52.5)	37 (60.7)	70 (69.3)	105 (76.1)	236 (66.7)	< 0.001^†^
	No	11 (78.6)	19 (47.5)	24 (39.3)	31 (30.7)	33 (23.9)	118 (33.3)	
Breastfeeding at night	None	2 (14.3)	9 (22.5)	8 (13.1)	22 (21.8)	28 (20.3)	69 (19.5)	0.424^†^
	0–6 months	0 (0.0)	1 (2.5)	2 (3.3)	7 (6.9)	12 (8.7)	22 (6.2)	
	7–12 months	3 (21.4)	5 (12.5)	9 (14.8)	12 (11.9)	19 (13.8)	48 (13.6)	
	13–18 months	4 (28.6)	2 (5.0)	14 (23.0)	18 (17.8)	29 (21.0)	67 (18.9)	
	> 19 months	5 (35.7)	23 (57.5)	28 (45.9)	42 (41.6)	50 (36.2)	148 (41.8)	
Sweetened milk	Yes	2 (14.3)	7 (17.5)	13 (21.3)	17 (16.8)	24 (17.4)	63 (17.8)	0.949^†^
	No	12 (85.7)	33 (82.5)	48 (78.7)	84 (83.2)	114 (82.6)	291 (82.2)	
Age recorded in completed years (month equivalents in parentheses). *Pearson chi-square test. ^†^Fisher exact test.								

**Table 2 Table2:** Number of children with dmft and pufa, and mean scores of variables

Variable	n (%)	Mean ± SD (median; minimum–maximum)
dmft	273 (77.1)	5.77 ± 4.78 (6; 0–20)
dt	260 (73.4)	5.18 ± 4.75 (5; 0–20)
mt	28 (7.9)	0.17 ± 0.78 (0; 0–8)
ft	51 (14.4)	0.36 ± 1.09 (0; 0–7)
pufa	180 (50.8)	1.54 ± 2.11 (1; 0–10)
p	167 (47.1)	1.28 ± 1.78 (0; 0–9)
u	28 (7.9)	0.10 ± 0.39 (0; 0–4)
f	31 (8.7)	0.12 ± 0.42 (0; 0–2)
a	7 (1.9)	0.03 ± 0.19 (0; 0–2)


The relationship between demographic features, daily habits, and the presence of pufa is presented in Table 3. No statistical difference was noted between sexes regarding ECC and pufa presence (*P* = 0.399 and *P* = 0.673, respectively, χ^2^). A statistically significant difference was found between groups with regard to parental educational level, family income, and frequency of dental visits (Fisher exact test,* P* = 0.017; χ^2^, *P* = 0.005 and *P* = 0.007, respectively). The presence of pufa was observed in 53.3% of children, whereas 36.3% of children in low-income families had no pufa scores (*P* = 0.005). Additionally, the presence of pufa increased with age (*P* = 0.020) (Table 4). The mean pufa score also increased significantly with age (Kruskal-Wallis, *P* = 0.037).

**Table 3 Table3:** Relationship between demographic features, daily habits, and presence of pufa

Variable		n (%)	pufa = 0, n (%)	pufa > 0, n (%)	*P* value
Sex	Girl	179 (50.6)	86 (48.0)	93 (52.0)	0.673
	Boy	175 (49.4)	88 (50.3)	87 (49.7)	
Educational level of parent	None	10 (2.8)	6 (3.5)	4 (2.2)	0.017*
	Primary–secondary	70 (19.8)	25 (14.5)	45 (24.7)	
	High school	122 (34.5)	56 (32.0)	66 (36.6)	
	University	152 (42.9)	87 (50.0)	65 (36.1)	
Family income	Low	159 (44.9)	63 (36.3)	96 (53.3)	0.005*
	Medium	104 (29.4)	58 (33.3)	46 (25.6)	
	High	91 (25.7)	53 (30.4)	38 (21.1)	
Number of children	1	133 (37.6)	73 (41.9)	60 (33.3)	0.127
	2	163 (46.0)	77 (44.2)	86 (47.8)	
	3	51 (14.4)	23 (13.4)	28 (15.6)	
	4	7 (2.0)	1 (0.6)	6 (3.3)	
Frequency of toothbrushing	Once a day	160 (45.2)	81 (46.6)	79 (43.4)	0.861
	Twice a day	109 (30.8)	53 (30.4)	56 (31.9)	
	Irregular	85 (24.0)	40 (23.0)	45 (24.7)	
Dental visits	Yes	236 (66.7)	104 (59.8)	132 (73.3)	0.007*
	No	118 (33.3)	70 (40.2)	48 (26.7)	
Breastfeeding at night	None	69 (19.5)	29 (16.7)	40 (22.2)	0.366
	0–6 months	22 (6.2)	10 (5.7)	12 (6.7)	
	7–12 months	48 (13.6)	29 (16.7)	19 (10.6)	
	13–18 months	67 (18.9)	31 (17.8)	36 (20.0)	
	> 19 months	148 (41.8)	75 (43.1)	73 (40.5)	
Sweetened milk	Yes	63 (17.8)	30 (17.2)	33 (18.3)	0.788
	No	291 (82.2)	144 (82.8)	147 (81.7)	
**P* < 0.05 (chi-square test).					

**Table 4 Table4:** Prevalence of pufa and its individual components (p, u, f, a) according to age group (n = 354)

Age group	n	pufa > 0, n (%)	pufa score, mean ± SD	Pulpal involvement (p), n (%)	Ulceration (u), n (%)	Fistula (f),n (%)	Abscess (a), n (%)
1 year (12–23 mo)	14	1 (7.1)	0.36 ± 1.34	1 (7.1)	0 (0.0)	0 (0.0)	0 (0.0)
2 years (24–35 mo)	40	19 (47.5)	1.10 ± 1.48	19 (47.5)	3 (7.5)	1 (2.5)	0 (0.0)
3 years (36–47 mo)	61	33 (54.1)	1.48 ± 1.96	30 (49.2)	6 (9.8)	4 (6.6)	1 (1.6)
4 years (48–59 mo)	101	54 (53.5)	1.75 ± 2.24	49 (48.5)	5 (5.0)	11 (10.9)	4 (4.0)
5 years (60–72 mo)	138	73 (52.9)	1.66 ± 2.26	68 (49.3)	14 (10.1)	15 (10.9)	2 (1.4)
Total	354	180 (50.8)	1.54 ± 2.114	167 (47.1)	28 (7.9)	31 (8.7)	7 (1.9)
*P* value		0.020*	0.037^‡^	0.051*	0.451^†^	0.285^†^	0.497^†^
Percentages are within each age group. pufa > 0, presence of ≥ 1 clinical consequence of untreated caries. *Pearson chi-square test. ^†^Fisher exact test. ^‡^Kruskal–Wallis test.							

The mean values for the ECOHIS child subscale, ECOHIS family subscale, and total ECOHIS score were 6.61 ± 5.72, 4.71 ± 3.41, and 11.47 ± 8.36, respectively. No statistically significant sex differences were found for the ECOHIS child subscale, ECOHIS family subscale, or total ECOHIS score (*P* = 0.994, *P* = 0.326, and *P* = 0.986, respectively). The highest mean ± SD from the ECOHIS assessment for all children were observed in the questions about pain (1.24 ± 1.05) and difficulty eating (1.00 ± 1.20) in the child subgroup, and discomfort (2.15 ± 1.51) in the family subgroup. The lowest and highest scores in CIS were 0 and 26, with 44 and 2 children, respectively. The lowest and highest scores in FIS were 0 and 16, with 61 and 3 children, respectively. The highest total ECOHIS score was 42, recorded in one child, whereas the lowest was 0 in 26 children.

Evaluation of ECOHIS scores regarding pufa and severity of ECC is shown in Table 5. Significant positive correlations were observed between pufa and CIS, FIS, and total ECOHIS scores (r = 0.510, *P* = 0.001; r = 0.514, *P* = 0.001; r = 0.565, *P* = 0.001, respectively). CIS, FIS, and total ECOHIS scores differed significantly across the groups of no ECC, non-severe ECC, and S-ECC (*P* < 0.001 for all).

**Table 5 Table5:** ECOHIS scores according to pufa presence and ECC severity

Variable		ECOHIS child	*P* value	ECOHIS family	*P* value	ECOHIS total	*P* value
pufa	pufa = 0	4.01 ± 4.45 (3; 0–25)	< 0.001*	3.07 ± 3.07 (2; 0–16)	< 0.001*	7.16 ± 6.67 (5; 0–33)	< 0.001*
	pufa > 0	9.13 ± 5.69 (8; 0–26)		6.30 ± 2.95 (6; 0–16)		15.64 ± 7.69 (16; 0–42)	
ECC severity	ECC = 0	3.17 ± 3.18 (2; 0–15)^ab^	< 0.001**	2.19 ± 2.96 (1; 0–16)^de^	< 0.001**	5.38 ± 5.31 (4; 0–24)^gh^	< 0.001**
	Non-severe ECC	5.73 ± 4.91 (5; 0–22)^ac^		4.06 ± 3.03 (4; 0–12)^df^		10.17 ± 7.64 (9; 0–33)^gj^	
	S-ECC	8.57 ± 6.14 (8; 0–26)^bc^		6.16 ± 3.01 (6; 0–16)^ef^		14.81 ± 8.12 (14; 0–42)^hj^	
**P* < 0.05, Mann–Whitney U; ***P* < 0.05, Kruskal–Wallis. Values are expressed as mean ± SD (median; minimum–maximum). Superscript letters denote significant pairwise differences (Dunn test, Bonferroni): a, *P* = 0.003; b, *P* < 0.001; c, *P* = 0.001; d–j, *P* < 0.001.							

Statistically significant differences were detected between carious conditions and CIS, FIS, and total ECOHIS scores (Table 6). In Dunn test pairwise comparisons, Group 1 (no caries) differed significantly from both Group 2 (caries without pufa) and Group 3 (caries with pufa) across all ECOHIS groups (*P* < 0.001 for all comparisons).

**Table 6 Table6:** ECOHIS scores by carious condition groups

Variable	ECOHIS child	*P* value	ECOHIS family	*P* value	ECOHIS total	*P* value
ECC = 0	3.17 ± 3.18 (2; 0–15)^ab^	< 0.001*	2.18 ± 2.96 (1; 0–16)^cd^	< 0.001*	5.38 ± 5.31 (4; 0–24)^ef^	< 0.001*
ECC > 0 + pufa = 0	4.49 ± 4.81 (3; 0–25)^a^		3.76 ± 2.96 (3; 0–10)^c^		8.39 ± 6.94 (7; 0–33)^e^	
pufa > 0	9.18 ± 5.79 (8; 0–26)^b^		6.30 ± 2.94 (6; 0–16)^d^		15.68 ± 7.80 (16; 0–42)^f^	
**P* < 0.05, Kruskal–Wallis. Values are expressed as mean ± SD (median; minimum–maximum). Superscript letters denote significant pairwise differences (Dunn test, Bonferroni): a–f, *P* < 0.001.						

Table 7 indicates significant differences in ECOHIS subscale scores between children with and without pufa. All domain mean scores were higher for children with pufa > 0.

**Table 7 Table7:** ECOHIS subscale scores in children with and without pufa

Scale/subscale		pufa = 0	pufa > 0	*P* value
CIS	Total CIS	4.01 ± 4.451 (3; 0–25)	9.13 ± 5.69 (8; 0–26)	< 0.001*
	Child symptoms	0.77 ± 0.90 (0; 0–3)	1.67 ± 1.00 (2; 0–3)	< 0.001*
	Child function	2.08 ± 2.45 (1.5; 0–13)	4.35 ± 2.87 (4; 0–12)	< 0.001*
	Child psychological well-being	0.95 ± 1.30 (0; 0–5)	2.42 ± 1.83 (2; 0–8)	< 0.001*
	Self-image and social interaction	0.29 ± 0.88 (0; 0–6)	1.22 ± 1.70 (0; 0–6)	< 0.001*
FIS	Total FIS	3.07 ± 3.07 (2; 0–16)	6.30 ± 2.95 (6; 0–16)	< 0.001*
	Parental distress	2.58 ± 2.54 (2; 0–8)	4.60 ± 2.04 (5; 0–8)	< 0.001*
	Family function	0.66 ± 1.45 (0; 0–8)	2.20 ± 2.30 (2; 0–8)	< 0.001*
Total ECOHIS		7.16 ± 6.67 (5; 0–33)	15.64 ± 7.69 (16; 0–42)	< 0.001*
**P* < 0.05, Mann-Whitney U test. Values are expressed as mean ± SD (median; minimum–maximum).				

Multiple linear regression analysis showed that caries-related variables explained 28% of the variance in ECOHIS scores (R^2^ = 0.280, *P* < 0.001). Higher ECOHIS scores were strongly associated with the presence of pufa (B = 7.656, 95% CI 5.790 to 9.523, *P* < 0.001). Although children with at least one carious lesion (dt) or extracted tooth (mt) were 1.9 and 2.4 times more likely, respectively, to have higher ECOHIS scores, these associations did not reach statistical significance (B = 1.904, 95% CI −0.243 to 4.050, *P* = 0.082; B = 2.479, 95% CI −0.284 to 5.241, *P* = 0.078, respectively).

## Discussion

This cross-sectional study evaluated the influence of ECC and caries-related pulpal involvement on OHRQoL among Turkish preschool children. Children with greater ECC severity and pulpal involvement exhibited higher ECOHIS scores than caries-free children or those without pufa, consistent with findings from several populations.^2,13,21,22,25
^


The ECC prevalence observed in this study (77.1%) was comparable to previous reports of 70.4%^2^ and 74.8%,^12^ although markedly lower prevalences (6% to 26.2%) have also been reported.^7,9,21
^ The pufa prevalence (50.8%) fell within the wide range reported internationally, from 13.2% to 26.2% in Brazil^12,21
^ to 71.4% in India^25^ and 84% in the Philippines,^16^ but exceeded the 4.4% reported in Germany,^7^ likely reflecting that country’s universal free annual dental checkup programme. Pulpal involvement was the most frequent pufa component (47.1%), in agreement with previous reports that identified pulpitis as the predominant consequence of untreated caries.^2,15,16,17
^


Both pufa prevalence and severity increased with age, plausibly reflecting the cumulative, chronic progression of untreated caries.^9^ No sex differences were observed in ECC or pufa, consistent with some reports,^10,11
^ though Ozsin Ozler et al^17^ found boys to be 2.49 times more affected by pufa than girls. Maternal and paternal education levels, as well as family income, were inversely associated with pufa presence, in line with previous reports indicating that children whose parents had less than a high school education were 2.7 times more likely to have PUFA.^17^ Dental visit frequency was also associated with pufa presence, whereas Kamran et al^10^ found no such association. Frequency of toothbrushing was not associated with pufa presence in the present study, consistent with a previous report.^17^


ECOHIS scores increased significantly with ECC severity, corroborating earlier reports that greater caries burden is associated with a more pronounced negative effect on OHRQoL.^1,2,9,28
^ Mean ECOHIS scores were significantly higher in children with pufa (15.64 ± 7.69) than in those without (7.16 ± 6.67), in agreement with previously reported score ranges.^9,20,25
^ This pattern suggests that pufa, by capturing symptomatic and functional consequences of untreated caries rather than merely recording decayed, missing, or filled teeth, more directly reflects OHRQoL.^12,21,25
^ Notably, both caries without pufa and caries with pufa contributed significantly to OHRQoL decline in the present cohort, whereas earlier studies conducted in non-clinical settings (schools, immunisation days) found no significant association for caries without pulpal involvement.^6,21
^ This discrepancy may reflect the present authors’ clinic-based sampling, in which parents seeking care were likely already concerned about their child’s dental condition.

All domains of the CIS showed a strong effect among children with pufa, whereas another study reported comparatively higher mean scores across all domains in children with pufa > 0, except for self-image and social interaction;^25^ similar patterns have been described in the original ECOHIS validation and other cultural contexts.^8,9,18,24
^ Parents appeared more attuned to their children’s physical complaints, psychological effects, and functional limitations than to social impacts. In the present study, both the FIS and CIS domains showed strong effects overall, although some studies have identified family distress as the most affected domain.^8,9,13,18,24,25
^


The present clinical findings are consistent with parental reports, showing that children with a positive pufa score were 7.6 times more likely to experience significant OHRQoL deterioration, indicating that ECOHIS reliably captures the clinical burden of untreated dental disease; this is consistent with a previously reported 6.26-fold effect.^12^ Cavitated dentine lesions were associated with a 1.9-fold increase in ECOHIS scores, although this did not reach statistical significance, in agreement with earlier findings.^12^


This study has several limitations. Its cross-sectional design precluded assessment of OHRQoL responsiveness over time, warranting longitudinal research. Additionally, recruitment from a single free paediatric dental clinic serving predominantly children with caries limits the generalisability of these findings. Future studies recruiting from community settings, such as schools or immunisation centres, as in the Turkish study by Sakaryali et al,^23^ would allow more representative sampling and direct comparison across clinical and non-clinical populations.

## Conclusion

In conclusion, caries severity and an increase in the pufa index negatively impact OHRQoL in preschool children. Compared to dental caries alone, an elevated pufa index exerted a greater detrimental effect on the OHRQoL of both children and their families. Additionally, children from socioeconomically disadvantaged families were more likely to have higher pufa scores due to untreated decay in primary teeth. The use of the pufa/PUFA index may offer greater potential to improve oral and dental health assessment than the dmft/DMFT index alone.

The authors declare no conflict of interest and received no financial support for this study. AI-assisted tools, including ChatGPT (OpenAI, GPT-5.5 Thinking) and Grammarly, were used solely for language editing, grammar checking, formatting, and improving the clarity of the manuscript, and all scientific content, data, conclusions, and final decisions are solely the responsibility of the authors. This study was conducted in compliance with the Declaration of Helsinki and was approved by the Dokuz Eylul University Research Ethics Committee (approval number 2023/10-13).

## References

## References

[ref1] Abanto J, Carvalho TS, Mendes FM, Wanderley MT, Bönecker M, Raggio DP (2011). Impact of oral diseases and disorders on oral health-related quality of life of preschool children. Community Dent Oral Epidemiol.

[ref2] Alanzi A, Husain F, Husain H, Hanif A, Baskaradoss JK (2023). Does the severity of untreated dental caries of preschool children influence the oral health-related quality of life. BMC Oral Health.

[ref4] Bittencourt JM, Martins LP, Paiva SM, Pordeus IA, Martins-Júnior PA, Bendo CB (2021). Early childhood caries and oral health-related quality of life of Brazilian children: Does parents’ resilience act as moderator. Int J Paediatr Dent.

[ref5] Chen KJ, Gao SS, Duangthip D, Lo ECM, Chu CH (2019). Prevalence of early childhood caries among 5-year-old children: A systematic review. J Investig Clin Dent.

[ref6] Corrêa-Faria P, Daher A, Freire MDCM, de Abreu MHNG, Bönecker M, Costa LR (2018). Impact of untreated dental caries severity on the quality of life of preschool children and their families: A cross-sectional study. Qual Life Res.

[ref7] Grund K, Goddon I, Schüler IM, Lehmann T, Heinrich-Weltzien R (2015). Clinical consequences of untreated dental caries in German 5- and 8-year-olds. BMC Oral Health.

[ref8] Hashim AN, Yusof ZY, Esa R (2015). The Malay version of the Early Childhood Oral Health Impact Scale (Malay-ECOHIS): Assessing validity and reliability. Health Qual Life Outcomes.

[ref9] Jaggi A, Marya CM, Nagpal R, Oberoi SS, Kataria S, Taneja P (2019). Impact of early childhood caries on oral health-related quality of life among 4–6-year-old children attending Delhi schools: A cross-sectional study. Int J Clin Pediatr Dent.

[ref10] Kamran R, Farooq W, Faisal MR, Jahangir F (2017). Clinical consequences of untreated dental caries assessed using PUFA index and its covariates in children residing in orphanages of Pakistan. BMC Oral Health.

[ref11] Khanal S, Shah P, Khapung A (2022). Assessment of sequelae of untreated dental caries using PUFA index in children visiting a tertiary center in Kathmandu. J Coll Med Sci Nepal.

[ref12] Leal SC, Bronkhorst EM, Fan M, Frencken JE (2012). Untreated cavitated dentine lesions: Impact on children’s quality of life. Caries Res.

[ref13] Leelataweewud P, Jirarattanasopha V, Ungchusak C, Vejvithee W (2021). Psychometric evaluation of the Thai version of the Early Childhood Oral Health Impact Scale (Th-ECOHIS): A cross-sectional validation study. BMC Oral Health.

[ref14] Li S, Malkinson S, Veronneau J, Allison PJ (2008). Testing responsiveness to change for the Early Childhood Oral Health Impact Scale (ECOHIS). Community Dent Oral Epidemiol.

[ref15] Mehta A, Bhalla S (2014). Assessing consequences of untreated carious lesions using pufa index among 5–6-year-old school children in an urban Indian population. Indian J Dent Res.

[ref16] Monse B, Heinrich-Weltzien R, Benzian H, Holmgren C, van Palenstein Helderman W (2010). PUFA – An index of clinical consequences of untreated dental caries. Community Dent Oral Epidemiol.

[ref17] Ozsin Ozler C, Uzamis Tekcicek M, Ozdemir P, Guciz Dogan B (2018). Pufa index and related factors among 36- to 71-month-old children in Turkey: A cross-sectional study. Oral Health Prev Dent.

[ref18] Pahel BT, Rozier RG, Slade GD (2007). Parental perceptions of children’s oral health: The Early Childhood Oral Health Impact Scale (ECOHIS). Health Qual Life Outcomes.

[ref19] Peker K, Uysal Ö, Bermek G (2011). Cross-cultural adaptation and preliminary validation of the Turkish version of the Early Childhood Oral Health Impact Scale among 5–6-year-old children. Health Qual Life Outcomes.

[ref20] Randrianarivony J, Ravelomanantsoa JJ, Razanamihaja N (2020). Evaluation of the reliability and validity of the Early Childhood Oral Health Impact Scale (ECOHIS) questionnaire translated into Malagasy. Health Qual Life Outcomes.

[ref21] Reis PPG, Jorge RC, Guerra BMS, Soviero VM (2025). Impact of pulp involvement due to caries on oral health-related quality of life in Brazilian schoolchildren: A cross-sectional study. Eur Arch Paediatr Dent.

[ref22] Reis PPG, Jorge RC, Marañón-Vásquez GA, Fidalgo TKDS, Maia LC, Soviero VM (2025). Impact of clinical consequences of pulp involvement due to caries on oral health-related quality of life in children and adolescents: A systematic review and meta-analysis. Caries Res.

[ref23] Sakaryali D, Bani M, Cinar C, Alacam A (2019). Evaluation of the impact of early childhood caries, traumatic dental injury, and malocclusion on oral health-related quality of life for Turkish preschool children and families. Niger J Clin Pract.

[ref24] Scarpelli AC, Oliveira BH, Tesch FC, Leão AT, Pordeus IA, Paiva SM (2011). Psychometric properties of the Brazilian version of the Early Childhood Oral Health Impact Scale (B-ECOHIS). BMC Oral Health.

[ref25] Sharna N, Ramakrishnan M, Samuel V, Ravikumar D, Cheenglembi K, Anil S (2019). Association between early childhood caries and quality of life: Early Childhood Oral Health Impact Scale and pufa index. Dent J (Basel).

[ref26] Sischo L, Broder HL (2011). Oral health-related quality of life: what, why, how, and future implications. J Dent Res.

[ref27] Vasavan SK, Retnakumari N (2022). Assessing consequences of untreated dental caries using pufa/PUFA index among 6–12-year-old schoolchildren in a rural population of Kerala. J Indian Soc Pedod Prev Dent.

[ref28] Wong HM, McGrath CP, King NM, Lo EC (2011). Oral health-related quality of life in Hong Kong preschool children. Caries Res.

